# Travel Time Information on Signalized Arterials

**DOI:** 10.3390/s25071977

**Published:** 2025-03-22

**Authors:** Jinhwan Jang

**Affiliations:** Korea Institute of Civil Engineering and Building Technology, Goyang 10223, Republic of Korea; jhjang@kict.re.kr

**Keywords:** travel time, DSRC, outlier filtering, prediction, CNN, LSTM

## Abstract

Travel time information has become an essential component of everyday commuting. Without such information, schedule delays would increase, leading to inevitable losses in traveler utility. In Korea, dedicated short-range communication transponders that identify vehicles have been installed along signalized arterials to collect travel time data. By matching vehicle identifications at consecutive points, travel times can be measured. However, for travel time information to be effective, two types of data processing techniques are required: outlier filtering and travel time prediction. This study proposes algorithms to address both challenges. An outlier filtering algorithm based on the median-based confidence interval was developed, taking into account the travel time characteristics on suburban arterials with frequent entry and exit points. Additionally, a travel time prediction algorithm that integrates Long Short-Term Memory (LSTM) and Convolutional Neural Networks (CNNs), referred to as LSTM-CNN, was developed to capture both long-term trends and local patterns in travel time data. The implementation of these algorithms resulted in a 2.2% reduction in error rates under congested conditions compared to current practices. At the 4 km study site, the annual benefits from this error reduction could amount to USD 135,200.

## 1. Introduction

Travel time information has become an essential aspect of daily life. Without access to such information, social utility may diminish due to early or late arrivals, leading to wasted time that could otherwise be allocated to more valuable activities. To collect travel time data, two types of traffic detectors have been deployed: point detectors and section detectors. Point detectors measure vehicle speed at a specific location, and travel time is estimated by dividing the distance by the measured speed. Section detectors, on the other hand, identify vehicles at distinct locations and match vehicle identifications at two consecutive points to calculate travel time. Point detectors have been recognized as effective for uninterrupted facilities, but they present limitations on interrupted facilities due to delays at intersections. Consequently, section detectors, which can directly measure travel times, have been installed on signalized arterials. As of 2021, 270 dedicated short-range communication (DSRC) transponders that identify passing vehicles were deployed on suburban arterials in Korea [1]. However, travel time information in DSRC systems on signalized arterials faces two main challenges: outlying travel time data and time lags in the collected data.

Outlying observations primarily occur due to exit and re-entry maneuvers between section detectors. Signalized arterials typically feature frequent intersections and roadside businesses along the route, which can lead to frequent exit and re-entry maneuvers. If these outlying observations are not properly addressed, the travel time information may become unreliable. Moreover, as travel time data are collected when vehicles complete their trips within section detector systems, the recorded travel times inherently exhibit a time lag. This lag renders the information less useful for drivers who are beginning their trips along the route. To address the issue of outliers, an outlier filtering algorithm needs to be developed, and to mitigate the time lag, a prediction technique must be applied. Currently, a median filter and k-Nearest Neighbor (k-NN) techniques are employed in the DSRC system. However, the median filter alone is insufficient for identifying all valid travel times, and the k-NN algorithm has demonstrated limitations in predicting travel times under congested conditions. Therefore, further improvements are necessary to enhance the effectiveness of the DSRC-based traffic information system.

In this study, two key challenges were addressed. To resolve the outlier issue, a median-based confidence interval concept was derived. To mitigate the time-lag phenomenon, a deep learning model combining Long Short-Term Memory (LSTM) and Convolutional Neural Networks (CNNs) was proposed. The proposed techniques were compared to the current methods, which rely on a simple median filter for outlier filtering and k-Nearest Neighbor (k-NN) for travel time prediction. Finally, the superiority of the developed techniques over the existing practices was thoroughly discussed.

## 2. Previous Research

### 2.1. Outlier Filtering

Numerous studies have been conducted to filter outlying travel times from valid data sets. In the early stages, outliers were removed using a mean-based threshold approach, where valid travel times in subsequent aggregation intervals were defined within a specific range based on the mean travel time of the previous interval [2,3,4]. However, this simple threshold scheme did not accurately capture the full range of travel time patterns, leading to the exclusion of valid travel times when the threshold was too low and the inclusion of outliers when the threshold was too high. To address the limitations of the mean-based threshold, more complex algorithms were proposed [5,6,7]. However, these sophisticated algorithms have shown limitations regarding real-time application, and the need to estimate multiple parameters has hindered their practical use in real-world systems.

The DSRC system examined in this study currently employs a median filter, where the average travel time is estimated using the median value within each aggregation interval. The median filter has demonstrated advantages in terms of practicality and reliability over conventional algorithms; however, it is still unable to identify all valid travel times. To analyze travel time patterns or distributions in greater detail, it is essential to obtain all valid individual travel times. Therefore, there is a need to develop a robust outlier filtering algorithm that not only identifies each valid travel time but also ensures practical applicability.

### 2.2. Travel Time Prediction

In the early stages, several techniques—including the Kalman filter, nonparametric time series analysis models, regression analysis, and k-NN models—were employed to forecast travel times for real-time traveler information [8,9,10,11]. Among these, k-NN models were found to be particularly effective for travel time prediction up until the mid-2010s [12,13,14,15,16,17]. However, the need for k-NN models to identify the k-nearest neighbors each time new data are received posed a significant challenge for real-time applications. Additionally, the computational resources required to operate the k-NN algorithm were substantial compared to other models.

In the late 2010s, deep learning models garnered significant attention in the field of travel time forecasting. Numerous pioneering studies investigated the application of LSTM and sequence-to-sequence (seq2seq) models, revealing that their performance substantially outperforms that of conventional models [18,19,20,21,22,23,24,25,26]. However, these studies primarily utilized individual deep learning models, suggesting that the integration of composite models could further enhance predictive accuracy.

The DSRC system examined in this study employs a k-NN model for travel time prediction. Acknowledging the limitations of k-NN models and the emergence of more advanced deep learning techniques, the operators of the DSRC system have been exploring artificial intelligence models to enhance the reliability of travel time predictions. In this context, an innovative deep learning model that integrates two architectures (CNN and LSTM) has been developed.

## 3. Methodology

### 3.1. Outlier Filtering

As discussed earlier, to address the current issues with outlier treatment—ensuring both practicality and the inclusion of all valid travel times—a robust outlier filtering algorithm (Equations (1)–(4)) based on the concept of confidence intervals was proposed. In determining the confidence interval, the median, rather than the mean, was utilized and adjusted with correction factors to account for the travel time data’s tendency to deviate abnormally from the valid values. This filtering algorithm effectively identifies all valid data points that fall within the established confidence interval.

(1) $$T_{AB}\left(t\right)=\frac{\sum_{i}\left(t_{B,i}-t_{A,i}\right)}{N\left(S_{AB}\left(t\right)\right)},\;\;\;where\;i\in S_{AB}(t)$$

(2) $$S_{AB}\left(t\right)\equiv \left\{k|t-T_{w}<t_{B,k}\leq t\right\}\cap \left\{m\left|{\tilde{x}}_{t}-z_{\alpha /2}SE<{(t}_{B,m}-t_{A,m}\right)\leq {\tilde{x}}_{t}+z_{\alpha /2}SE\right\}$$

(3) $${\tilde{x}}_{t}=median(t_{B,m}-t_{A,m})$$

(4) $$SE=[1.253\times (\frac{\left(Q3-Q1\right)}{1.35})]/\sqrt{n}$$

where

ㆍ *T_AB_*(t) = average of valid travel times from A to B at time t;
ㆍ N = number of samples in 5 min block of travel times;
ㆍ *S_AB_*(t) = set of valid travel times from A to B at time t;
ㆍ *T_W_* = aggregation (collection) interval;
ㆍ *t_A_*_(*or B*),*i*(*or m*)_ = detection time of vehicle i (or m) at point A (or B);
ㆍ 1.253 and 1.35 = conversion factors (refer to [27,28,29]).

The developed filtering algorithm is practically applicable to real-world systems, as it does not require the estimation of the complex parameters used in previous studies. Typically, the aggregation interval is set at 5 min. During each five-minute interval, the median and standard error (SE) of all travel times recorded by traversing vehicles are calculated, and a confidence interval (e.g., 95%, 99%) is derived based on these values. Finally, individual travel times falling within this confidence interval are identified as valid data.

### 3.2. Travel Time Prediction

**Figure 1:**
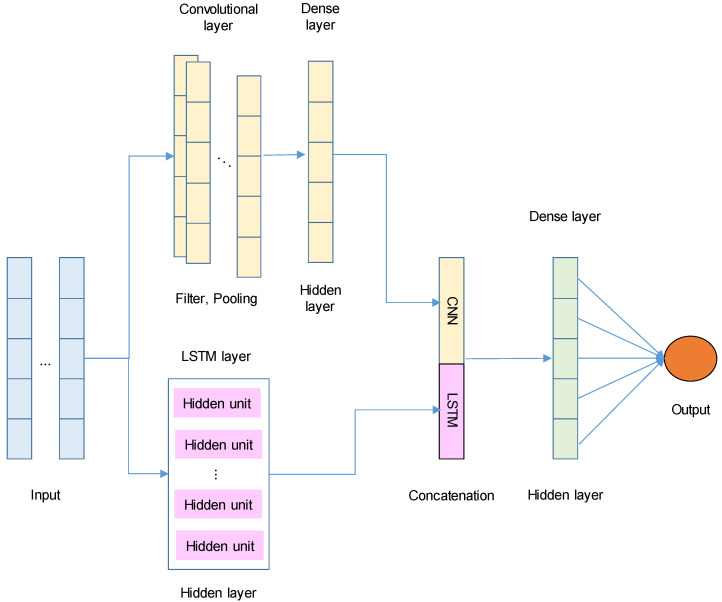
LSTM-CNN architecture.

As previously described, various deep learning models have been employed to forecast travel times. However, most prior studies have predominantly utilized a single model. In this study, a hybrid model was proposed to effectively capture both long-term and local patterns in travel time data, thereby enhancing prediction accuracy. The proposed Long Short-Term Memory–Convolutional Neural Network (LSTM-CNN) model integrates an LSTM network (Equations (5)–(10)) with CNNs (Equation (11)). This architecture is designed to capture temporal dependencies through LSTMs and spatial or local patterns through CNNs in the time-series travel time data. The architecture of the LSTM-CNN model is depicted in Figure 1. Both components operate independently to learn temporal and local patterns, respectively, followed by a concatenation layer to integrate their outputs.

In an LSTM network, the key equations govern the behavior of the gates (forget, input, and output) and the cell state. The LSTM equations are as follows:

(5) $$f_{t}=\sigma W_{f}\cdot x_{t}+U_{f}\cdot h_{t-1}+b_{f}$$

(6) $$i_{t}=\sigma (W_{i}\cdot x_{t}+U_{i}\cdot h_{t-1}+b_{i})$$

(7) $${\tilde{C}}_{t}=tanh(W_{C}\cdot x_{t}+U_{C}\cdot h_{t-1}+b_{C})$$

(8) $$C_{t}=f_{t}⊙C_{t-1}+i_{t}⊙{\tilde{C}}_{t}$$

(9) $$o_{t}=\sigma (W_{o}\cdot x_{t}+U_{o}\cdot h_{t-1}+b_{o})$$

(10) $$h_{t}=o_{t}⊙tanh(C_{t})$$

where

ㆍ *x_t_* = input at time step t;
ㆍ $h_{t-1}$ = hidden state from the previous time step;
ㆍ $C_{t-1}$ and $C_{t}$ = previous and present cell states;
ㆍ $f_{t},i_{t},o_{t}$ = forget, input, and output gates;
ㆍ ${\tilde{C}}_{t}$ = candidate cell state;
ㆍ $W_{f},W_{i},W_{C},W_{o}$ = input weights for the gates and cell state;
ㆍ $U_{f},U_{i},U_{C},U_{o}$ = recurrent weights;
ㆍ $b_{f},b_{i},b_{C},b_{o}$ = biases;
ㆍ σ and tanh = sigmoid and hyperbolic tangent functions;
ㆍ ⊙ = element-wise multiplication.

In a CNN, the convolution operation is fundamental. The convolution process can be represented by the following equation:

(11) $$Z_{i,j,k}=\sum_{m=0}^{M-1}\sum_{n=0}^{N-1}X_{i+m,j+n,c}\cdot W_{m,n,c,k}+b_{k}$$

where

ㆍ $Z_{i,j,k}$ = output feature map at position (i,j) and channel k;
ㆍ $X_{i+m,j+n,c}$ = input value at position (i+m,j+n) for channel c;
ㆍ $W_{m,n,c,k}$ = convolution filter weights;
ㆍ $b_{k}$ = bias term for channel k;
ㆍ M and N = dimensions of the filter and kernel.

The benefits of the proposed LSTM-CNN model over the previous models that exclusively utilized a single model are as follows:

- Temporal and spatial dependencies: LSTM effectively captures long-term temporal dependencies, while CNN extracts spatial or local patterns from the data.
- Multi-scale learning: CNN captures patterns at multiple scales through convolutional filters, aiding in the detection of important features in time-series data.
- Dimensionality reduction: pooling layers in CNN reduce dimensionality and computational complexity, thereby enhancing model efficiency.

## 4. Study Site

**Figure 2:**
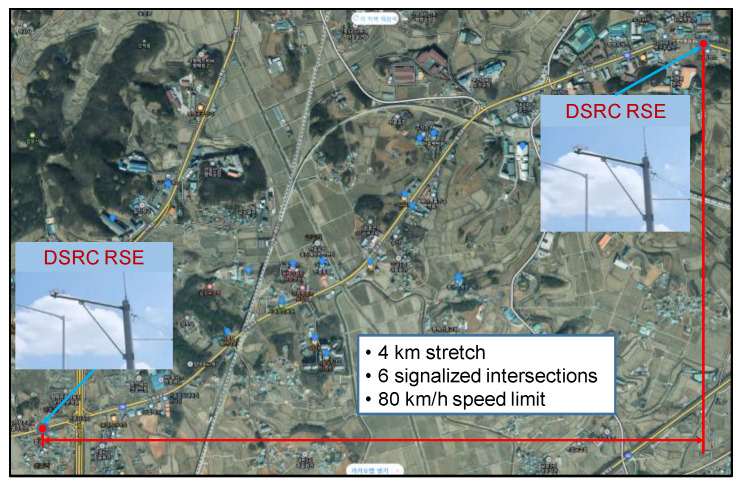
Experiment segment.

To evaluate the developed algorithms, travel time data were collected from a DSRC-based traffic information system deployed on National Highway 38 in the Pyeongtaek region of South Korea. As illustrated in Figure 2, the study section spans 4 km and comprises a total of six signalized intersections. DSRC Roadside Equipment (RSE) was installed at both ends of the section to record the passing times of vehicles equipped with a DSRC On-Board Unit (OBU). As of the time the experimental data were collected, approximately 60% of vehicles in Korea were equipped with DSRC OBUs.

Despite the posted speed limit of 80 km/h, the average speed during non-congested periods was around 50–60 km/h due to the high density of traffic signals along the route. The area is situated near an industrial complex, leading to congestion during weekday morning commutes. Since there is no congestion on weekends due to the lack of commuter traffic, the data collection focused on weekdays in January 2013, when morning congestion was consistently observed.

**Figure 3:**
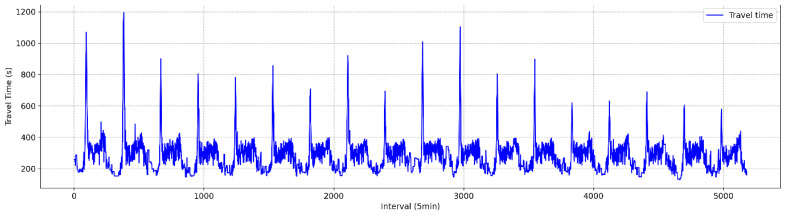
Baseline travel times (5 min aggregation interval mean).

The baseline data for the evaluation were generated by the operators of the DSRC system. These operators manually identified valid travel times based on their prior knowledge of the section and monitoring results from surveillance cameras installed along the route. The baseline data verified by the operators are depicted in Figure 3, and the descriptive statistics are presented in Table 1.

The travel time data were aggregated at 5 min intervals, in alignment with the DSRC-based traffic information system deployed on the National Highway. Daily congestion was observed between 8:00 and 9:00 a.m., whereas in the afternoon, although traffic volume increased, the rise in travel time was less pronounced compared to the morning peak period. A total of 5181 data points were collected at 5 min intervals over approximately 18 days. As indicated in Table 1, the average travel time for the study section was 286 s, with a standard deviation of 106 s, a minimum of 130 s, and a maximum of 1193 s. The maximum travel time was nearly five times the average, highlighting significant congestion during morning peak hours.

## 5. Generation of Travel Time Information

### 5.1. Outlier Filtering

**Figure 4:**
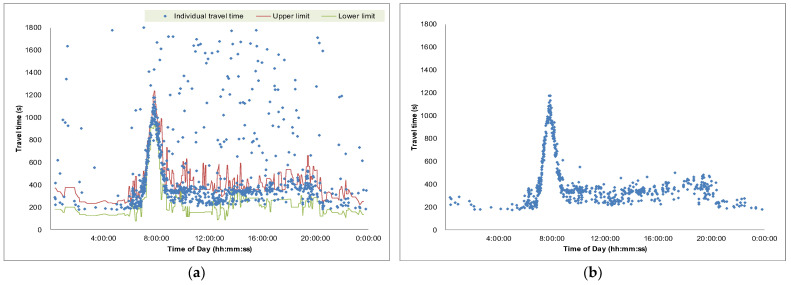
Individual travel time: (**a**) raw data, (**b**) outlier-filtered data.

The proposed outlier filtering algorithm was applied to the collected weekday travel time data described previously. A one-day example is illustrated in Figure 4, demonstrating that all apparent outliers were successfully filtered after applying the algorithm. As mentioned earlier, the current practice generates only one median value per aggregation interval (5 min), which prevents the identification of individual travel time records. In contrast, when the developed algorithm is applied, all individual travel time records can be retrieved, as shown in Figure 4b.

The algorithm’s capability to capture the entire set of valid travel times allows practitioners to gain deeper insights into travel time patterns. For example, it reveals that the variability of travel times under congested conditions is relatively small compared to that under uncongested conditions. This is likely attributable to the nature of interrupted flow facilities, where travel times tend to vary more widely under free-flow conditions due to traffic signal effects. In contrast, during congestion, the influence of signals diminishes as vehicles queue. Leveraging this understanding, operators could refine travel time information dissemination strategies, such as providing a range of travel times instead of a single average value. Additionally, by obtaining individual travel times, valuable traffic statistics—such as travel time reliability and various descriptive measures—can be derived, which are otherwise unattainable using the current practice.

### 5.2. Travel Time Prediction

The proposed LSTM-CNN algorithm was implemented using the *TensorFlow Keras* framework. The filtered travel time data were standardized using the *MinMaxScaler* to improve prediction performance. A target prediction interval of 30 min was selected. No significant differences in performance were observed when varying the prediction target from 10 min to 1 hour. Consequently, the feature columns included travel times at both the current time and 30 min ahead, while the label column represented the travel time at 30 min ahead. The window size for time series analysis was set to 288 (5 min * 288 = 24 h) to capture the recurrent daily travel time pattern. The processed data were then split into a 7:3 ratio for training and testing.

**Figure 5:**
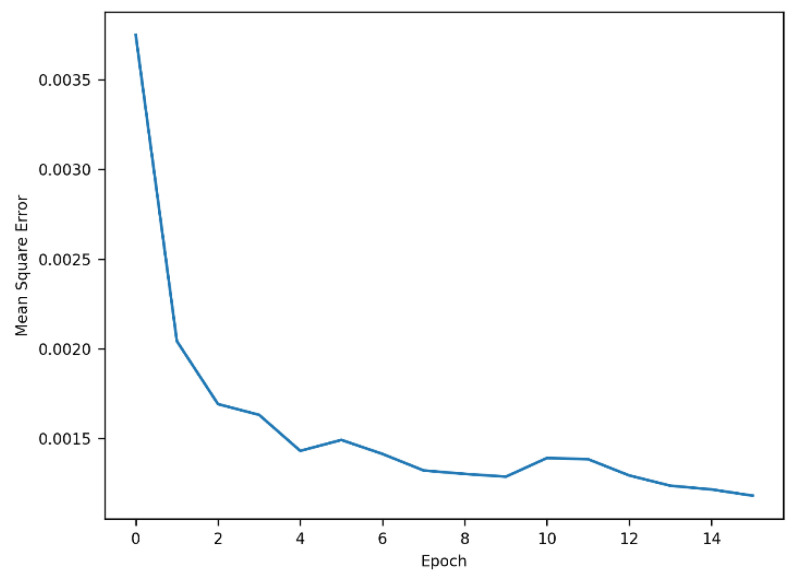
Learning process of LSTM-CNN.

The grid search technique was employed to identify the optimal parameters. Table 2 presents the parameters explored and the corresponding optimal values determined for each model. To prevent overfitting, the *EarlyStopping* function with a patience of five was applied, restoring the best weights where the mean square error was minimized. Additionally, a dropout rate of 0.3 was applied to the final dense layer. The training process, as depicted in Figure 5, shows that the reduction in error became relatively small after four epochs.

## 6. Evaluation

The forecasted travel times were evaluated using three widely recognized metrics (Equations (12)–(14)): Mean Absolute Error (MAE), Root Mean Square Error (RMSE), and Mean Absolute Percentage Error (MAPE). MAE, the mean of absolute errors, is intuitively easier to interpret compared to RMSE. However, RMSE assigns greater weight to larger errors than MAE, often resulting in higher values. According to a study [30], MAE is more appropriate when errors follow a normal distribution, whereas RMSE is better suited for errors following a Laplace distribution. Therefore, both measures are typically employed to assess prediction performance. MAPE converts MAE into a percentage, facilitating comparison across different scales and improving interpretability.

(12) $$MAE=\frac{\sum \left|y-\hat{y}\right|}{n}$$

(13) $$RMSE=\sqrt{\frac{\sum {\left(y-\hat{y}\right)}^{2}}{n}}$$

(14) $$MAPE=\frac{100}{n}\sum \left|\frac{y-\hat{y}}{y}\right|$$

where

ㆍ y = observed value;
ㆍ $\hat{y}$ = predicted value;
ㆍ n = number of samples.

**Figure 6:**
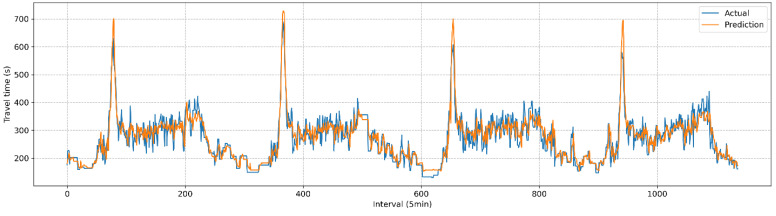
Comparison of predicted travel time and actual travel time (current).

**Figure 7:**
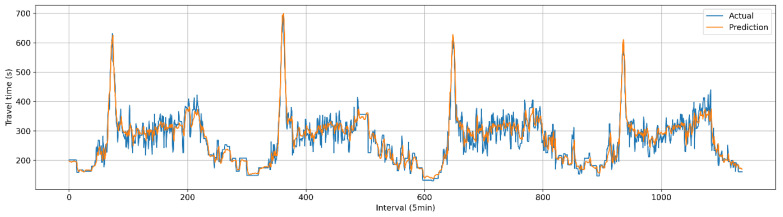
Comparison of predicted travel time and actual travel time (proposed).

**Figure 8:**
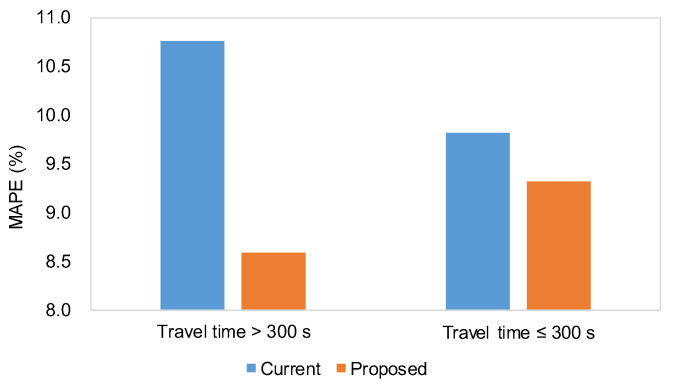
Travel time error differences between models by flow condition.

The predicted travel times generated by the current and proposed techniques were evaluated using the baseline data described in Figure 3. Table 3 presents the results for the three metrics: MAE, RMSE, and MAPE. The performance of the proposed methodology demonstrated a slight improvement (1.3%) over the current practice. Although the improvement was modest, it was found to be statistically significant, as determined by a paired *t*-test at a significance level of 0.05 (see Table 4). Figure 6 and Figure 7 depict the comparisons between actual and predicted travel times for the current and proposed algorithms. Given the heightened emphasis on travel time information under congested conditions, the prediction performances were categorized into congested and non-congested conditions, with a threshold of 300 s (1.5 times the free-flow travel time of 200 s). The results indicated the performance improvement (2.2%) of the proposed method was more pronounced under congested conditions, as shown in Table 5 and Figure 8.

**Figure 9:**
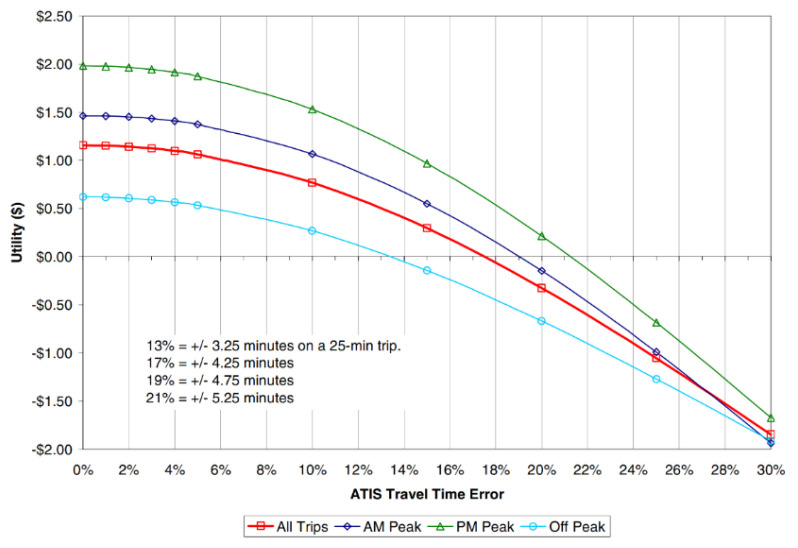
Traveler utility as a function of travel time error (source: [31]).

Estimating the improvement in prediction performance in monetary terms could provide valuable insights. For this purpose, the travel time information utility function developed by Toppen was employed. The underlying logic of the utility function, based on data collected in Los Angeles, is that reduced schedule delays result from more accurate travel time information. Schedule delays occur when travelers arrive either earlier or later than expected due to inaccurate travel time information. This suggests that reliable travel time information increases traveler utility by enabling them to use their time more productively. Figure 9 presents the traveler utility curve developed by Toppen [31]. According to the graph, a 2.2% reduction in error during the morning peak corresponds to a utility increase of USD 0.20 per person. When applied to the average traffic volume and passenger occupancy on the 4 km stretch of the study site, this could translate to an approximate annual social benefit of USD 135,200.

- USD 0.20 * 2000 vehicles * 1.3 passengers * 5 days * 52 weeks = USD 135,200

## 7. Conclusions and Future Studies

Real-time travel time information is a critical element in our daily life. Despite numerous studies conducted over the past several decades, drivers continue to demand more reliable travel time data. To address this need, advanced data processing techniques for DSRC-based traffic information systems have been developed. Prior to this development, current practices and previous studies were thoroughly reviewed, with their limitations identified. These findings underscore the necessity for further advancement in algorithms for outlier filtering and travel time forecasting.

The developed outlier filtering algorithm is grounded in the concept of a median-based confidence interval. Appropriate statistical modifications were applied to the median to utilize the confidence interval of the normal distribution. The proposed technique was implemented on raw travel time data collected from a signalized arterial and demonstrated its effectiveness in censoring outliers from valid travel times. In contrast to the current practice, where a single median value is extracted for every 5 min aggregation interval, the developed algorithm retrieves the entire set of valid data. This advancement allows operators to acquire more detailed insights into travel time patterns, enhancing travel time information provision strategies and statistical analyses.

To address the time-lag effect in DSRC-based traffic information systems, a hybrid model combining LSTM and CNN architectures, referred to as the LSTM-CNN model, was proposed. This deep learning model demonstrated superior performance, achieving a 2.2% reduction in error compared to the traditional k-NN algorithm when applied to outlier-filtered travel time data. Unlike previous studies that employed only a single deep learning model, the proposed hybrid model captures both long-term and local patterns simultaneously. Furthermore, the performance improvement was quantified in monetary terms, yielding an estimated annual social benefit of USD 135,200 on the 4 km experimental stretch.

While the developed algorithms have been rigorously tested on the experimental section characterized by recurrent congestion, further application to travel time data from diverse arterial roads is necessary to ensure their generalizability. Additionally, more advanced deep learning models could be employed to achieve further error reduction, ultimately enhancing traveler utility. Though the proposed prediction algorithm exhibited satisfactory outcomes for recurrent congestion, further verification using travel time data for non-recurrent congestion during incidents or special holidays needs to be performed. Additionally, the developed outlier filtering algorithm still missed a few outliers (Figure 4b) that should have been censored. Future research is necessary for further improvement.

## Figures and Tables

**Table 1:** Descriptive statistics of travel time.

Statistic	Travel Time (s)
Count	5181
Mean	286
Standard deviation	106
Minimum	130
Maximum	1193

**Table 2:** LSTM-CNN parameters.

Model	Parameter	Search Grid	Optimal Parameter
LSTM	Number of hidden nodes	[64, 128, 256]	128
	Activation function	[Sigmoid, Tanh]	Tanh (hyperbolic tangent)
	Window size	[144, 288, 576]	288 (24 h)
CNN	Number of layers	[1, 2, 3]	2
	Activation function	[Sigmoid, ReLU]	ReLU

**Table 3:** Travel time error.

Measure	Current ^a^	Proposed ^b^
MAE (s)	27.3	23.9
RMSE (s)	37.3	32.3
MAPE (%)	10.3	9.0

^a^ median + k-NN; ^b^ proposed outlier filtering algorithm + LSTM-CNN.

**Table 4:** Paired *t*-test of MAPE.

Statistic	Current	Proposed
Mean	10.3	9.0
Variance	83.6	70.3
Number of samples	1137	
t-statistic	3.52	
t-statistic at sig. lev. of 0.05	1.65	
*p*-value (one-sided)	0.0002	

**Table 5:** Paired *t*-test of MAPEs by flow condition.

Statistic	Travel Time > 300 s		Travel Time ≤ 300 s	
	Current	Proposed	Current	Proposed
Mean	10.8	8.6	9.8	9.3
Variance	99.6	65.7	76.7	51.9
Number of samples	388		749	
t-statistic	4.62		0.93	
t-statistic at sig. lev. of 0.05	1.65		1.65	
*p*-value (one-sided)	2.27 × 10^−6^		0.18	

## Data Availability

Data are contained within the article.
